# Normative equations for central augmentation index: assessment of inter-population applicability and how it could be improved

**DOI:** 10.1038/srep27016

**Published:** 2016-05-27

**Authors:** Ana Jeroncic, Grgo Gunjaca, Danijela Budimir Mrsic, Ivana Mudnic, Ivica Brizic, Ozren Polasek, Mladen Boban

**Affiliations:** 1 Department of Research in Biomedicine and Health, University of Split School of Medicine, Split, Croatia; 2 Department of Pharmacology, University of Split School of Medicine, Split, Croatia; 3 Department of Pharmacology, University of Mostar School of Medicine, Mostar, Bosnia and Herzegovina; 4 Department of Public Health, University of Split School of Medicine, Split, Croatia

## Abstract

Common reference values of arterial stiffness indices could be effective screening tool in detecting vascular phenotypes at risk. However, populations of the same ethnicity may differ in vascular phenotype due to different environmental pressure. We examined applicability of normative equations for central augmentation index (cAIx) derived from Danish population with low cardiovascular risk on the corresponding Croatian population from the Mediterranean area. Disagreement between measured and predicted cAIx was assessed by Bland-Altman analysis. Both, cAIx-age distribution and normative equation fitted on Croatian data were highly comparable to Danish low-risk sample. Contrarily, Bland-Altman analysis of cAIx disagreement revealed a curvilinear deviation from the line of full agreement indicating that the equations were not equally applicable across age ranges. Stratification of individual data into age decades eliminated curvilinearity in all but the 30–39 (men) and 40–49 (women) decades. In other decades, linear disagreement independent of age persisted indicating that cAIx determinants other than age were not envisaged/compensated for by proposed equations. Therefore, established normative equations are equally applicable to both Nordic and Mediterranean populations but are of limited use. If designed for narrower age ranges, the equations’ sensitivity in detecting vascular phenotypes at risk and applicability to different populations could be improved.

Arterial stiffness, a well established risk factor for cardiovascular morbidity and mortality^1^,^2^, can be assessed non-invasively by several approaches. Carotid-femoral pulse wave velocity (PWV) measurement is considered as the “gold standard” method directly related to the arterial stiffness. Pulse wave analysis (PWA) provides several complementary indices that are indirectly associated with arterial stiffness^3^. Among them, the central augmentation index (cAIx) is widely used as an index of pulse wave reflection that significantly influences the central pressure profile, and has been shown to be an independent predictor of cardiovascular events (Fig. 1)^4^,^5^. The cAIx is usually measured by applying a generalized transfer function to the radial pressure waveform. It is an easily obtainable parameter that is both technically less demanding and less time consuming than the PWV measurement, and as such more convenient for daily clinical practice.

Although cAIx is a composite measure whose determinants are still matter of debate^6^, it is largely influenced by the individuals’ age, sex, height, and heart rate^7^,^8^,^9^. There are large inter-individual variations of cAIx values, due to the variability in physiological determinants of cAIx, which limits its clinical usefulness in identifying patients at risk or establishing it as a target for therapy.

In order to help individuals’ hemodynamic assessment several studies offered reference cAIx values based on the data from ethnically diverse populations^9^,^10^,^11^,^12^. However, a challenge could arise when comparing a cAIx value measured in an individual of certain anthropometric characteristics with the reference data representing average population values adjusted to various physiological determinants of cAIx.

As a further step in dealing with this issue, two studies proposed novel sex-13 and ethnic-specific^14^ normative equations that allow a standardized comparison between individual augmentation index (AIx) measurements and the calculated reference value, taking into account the subjects’ age, body height, and heart rate. The study by Chirinos *et al.* showed that besides the aforementioned physiological determinants of AIx, ethnic background also appears to be an independent determinant of the pulse wave reflection^14^. Janner *et al.* generated a new sex – specific internally validated equation adjusted for age, heart rate, and height in order to calculate reference values of cAIx that can be used to complement the interpretation of individual hemodynamic assessments among men and women in the Danish population with a low cardiovascular risk^13^.

The predictive equations from these two studies, unfortunately, cannot be directly compared because of differences in the method of computing AIx and the selection criteria of the reference sample from which normative data were acquired.

Both studies, however, opened the question on the potential effect of the living environment on population reference values of cAIx. Whether the proposed equations based on data from the Danish population can equally be applied to a population of a similar ethnic background from a different geographic region, life style, and cultural heritage remains to be answered. In this work, we focused on Danish normative equations, since we used the same measuring device, method of calculating cAIx, as well as the same inclusion/exclusion criteria in selecting a cohort with a low cardiovascular risk.

Hence, the first aim of this study was to validate the applicability of the proposed normative equations from the Danish population to the matching Croatian population living in the Mediterranean region of Croatia.

The second aim was to establish whether the proposed equations equally apply across age ranges. Namely, the proposed “Danish” equations allow a rather wide prediction interval for reference cAIx. Considering that the age is the most important determinant of cAIx, and that hemodynamic changes across ages differently inter-relate with arterial stiffness indices^15^,^16^, we tested applicability of equations in different age decades of the examined population sample.

## Results

### Population-based sample

Baseline characteristics of the initial Croatian population-based sample that comprised 394 men (39%) and 614 women (61%) are presented in Table 1 along with the data on the Danish general population sample published by Janner *et al*.^13^.

### The low-risk cohort

The low risk Croatian cohort included 538 participants who fully complied with the Danish eligibility criteria: 148 men and 390 women. The mean ± SD of observed cAIx in the low risk cohort was: 22.5% ± 12.7 in women, and 12.1% ± 14.4 in men.

**Figure 2:**
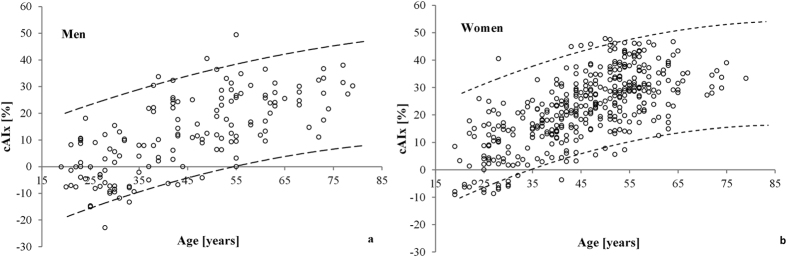
The relationship between the central augmentation index (cAIx) and age in the Croatian low risk cohort in (**a**) men and (**b**) women. The ninety-five percent prediction limits curves representing data from the Danish low risk cohort are superimposed onto Croatian individual data.

The cAIx *vs.* age distribution in the Croatian low risk cohort was highly comparable with the Danish low risk cohort as shown in Fig. 2. Namely, individual data from the Croatian low risk cohort almost completely fell within 95% prediction interval curves derived from the Danish low risk cohort. Besides high comparability of the two datasets, this finding also confirms successful replication of the eligibility criteria.

**Figure 3:**
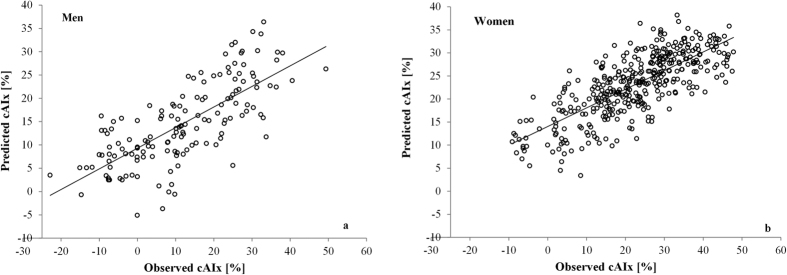
Scatterplots of predicted vs. observed cAIx values with correlations, shown for (**a**) men and (**b**) women.

When the Danish normative model was applied on the data from the low risk Croatian cohort no significant differences were found in regression coefficients for cAIx between original and fitted equations, indicating a similarity for reference cAIx between these two populations (Table 2). Moreover, a strong correlation between actually measured cAIx values in the Croatian cohort and those predicted by the Danish normative equations was found (r = 0.73 in men, and 0.76 in women, P < 0.001 for both; Fig. 3).

### Agreement of cAIx values observed in the Croatian low risk cohort with values predicted by the Danish normative model

**Figure 4:**
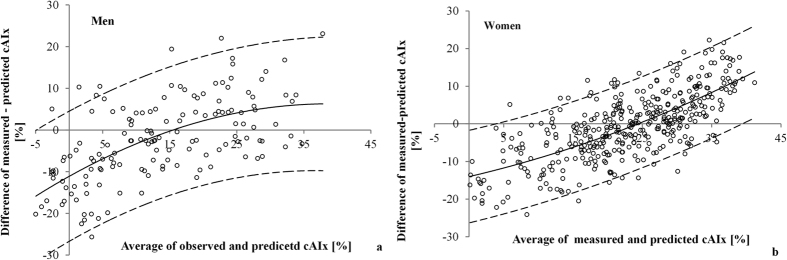
Bland-Altman plots showing agreement between central augmentation index (cAIx) values measured in the Croatian low risk cohort and those predicted by the Danish normative equation plotted against average cAIx value, for both (**a**) men and (**b**) women. Shown are: individual data from the Croatian low risk cohort (open circles), the fitted regression curve (full black lines), and 95% limits of agreement (dashed black lines).

When apparently good agreement in reference cAIx values between the Croatian and the Danish low risk cohorts shown in Fig. 2 was analyzed by Bland-Altman (BA) plots, intriguing differences emerged (Fig. 4).

By plotting differences between cAIx values observed in the Croatian cohort and those predicted by the Danish normative equations against the average cAIx value, a nonlinear deviation from the line of full agreement was found. At low average cAIx values, disagreement was high in a way that observed cAIx values were lower than predicted, whereas the opposite was observed for high average cAIx values, both in men and women. Furthermore, the analysis of BA plots identified non-linear regression curves as optimal models for describing the dependency of cAIx differences against averages (adjusted R^2^ of 0.39 in men and 0.52 in women, p for coefficients < 0.05). Although the variance around the fitted line was constant for both sexes (i.e., homoscedasicity, F-test on residuals, p = 0.99), the 95% limits of agreement were rather wide: ±15.5% cAIx in men and ±12.1% in women.

### The age effects on cAIx disagreement

**Figure 5:**
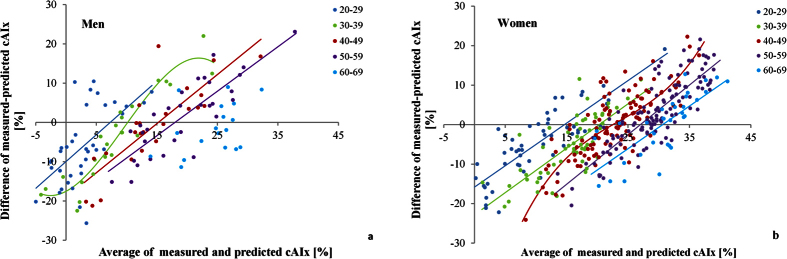
Bland-Altman plots on agreement between central augmentation index (cAIx) values measured in the Croatian low risk cohort and those predicted by the Danish normative equation, plotted against average cAIx value and stratified by age decades in (**a**) men, and (**b**) women. Colors correspond to different age decades. Dots of the same color represent individual data whereas thick lines in the matching color show the best fitting regression line in particular age decade. A regression line for men 60–69 years of age was omitted due to a small sample size in this age decade.

The age was the only determinant included in normative equations that significantly correlated with differences between measured and predicted cAIx in men (multiple regression analysis, beta_age_ = 0.296, P = 0.001) and women (beta_age_ = 0.259, P < 0.001; beta_age2_ = −0.151, P = 0.002). The curvilinear relationship between differences of measured and predicted cAIx (hereinafter referred to as cAIx disagreement) against cAIx averages in BA plots, indicates that the proposed normative equations for cAIx do not equally hold true across age ranges. Therefore, we repeated this analysis after stratifying individual data into age decades in order to analyze the age effect in more details. By doing so we obtained, in all but one age decade in men and women, a linear relationship of cAIx disagreement against cAIx averages, with approximately parallel shift between age groups (Fig. 5). This indicates that effect of age on observed disagreement between measured and predicted cAIx values was generally homogeneous. So, after analysis of the dependency of cAIx disagreement on age in different age decades, we found a non-significant effect of the age (ANOVAs for regression, p > 0.05). Only within the age decades of men aged 30–39 and women aged 40–49 years, a significant and nonlinear association of cAIx difference and age was observed (ANOVAs for regression, p from 0.031 to 0.043). Moreover, only in these two decades BA analysis showed a curvilinear relationship of cAIx disagreement against the cAIx averages (adjusted R^2^ from 0.63 to 0.75, p for coefficients < 0.05) (Fig. 5).

## Discussion

In this study, we performed an external validation of normative equations for cAIx derived from the Danish population with a low cardiovascular disease (CVD) risk by applying it to the matching Croatian population living in the Mediterranean region of Croatia. Although, it has been shown that environmental pressure may be an important determinant of vascular phenotype among people of the same ethnicity^17^,^18^, no study has examined the applicability of the normative equations established in one population on another population of the same ethnicity but of different environmental conditions and life style, by replicating the same normative model and using the same inclusion criteria and measuring device.

The Croatian and Danish low risk cohorts were apparently comparable regarding cAIx-age relationship as individual Croatian data were almost completely contained within the 95% prediction intervals of the cAIx from the Danish cohort (Fig. 2). Moreover, the distribution of individual data from the Croatian cohort was consistent with findings of previous studies analyzing the cAIx-age relationship, which showed that cAIx increases with age in a nonlinear manner with the tendency to level off after approximately 60 years of age^9^,^12^,^13^.

Replication of the Danish normative model on the individual Croatian dataset confirmed general applicability of the proposed Danish normative equations as the regression coefficients of the original and the fitted equations did not significantly differ. Based on these findings it could be concluded that the reference cAIx between these two low risk populations is highly similar.

This level of similarity appears to be satisfactory for studies establishing and comparing reference values for the augmentation index between different populations. However, it appears that stopping at that level of analysis may mask some important aspects of the normative equations exploitation.

Namely, the proposed normative equations still tolerate a rather wide range of individual variability in cAIx values as “normal”, which reduces their discriminative power in risk stratification and consequently, their clinical usefulness. Our results give additional support of this notion as we demonstrate that the proposed equations do not equally hold true across age ranges. This is best noticeable from the BA analysis showing that the difference in cAIx values actually measured in the Croatian cohort and those predicted by Danish normative equations nonlinearly deviated from the line of full agreement (Fig. 4). By analyzing disagreement between measured and predicted cAIx values at the level of age decades, we minimalized the effect of age, the most important determinant of cAIx, and yet the cAIx disagreement was still preserved in an intriguing fashion. The fact that the cAIx disagreement persisted despite the diminished age effect and that the disagreement was linearly related to the cAIx average, implies that factors other than age are not compensated for, or envisaged by the present normative equations. Although the proposal of a new normative equation is beyond the scope of this paper, we believe that normative equations for cAIx besides age, height, and heart rate should include other proven determinants of wave reflection parameters such as diastolic or mean arterial pressure^9^,^19^,^20^. An approximately parallel shift of the described linear disagreement between age decades indicates that the uncounted impact of other predictor(s) is largely preserved. In contrast to other age strata, in the age decades of 30–39 for men and 40–49 for women the impact of age was still significant resulting in a curvilinear relationship of cAIx disagreement vs average cAIx. Although we can only speculate on the physiological basis of this finding, it is indicative of the dynamic age-related structural/functional changes of the arterial tree occurring in these age groups. It is important to note that, regardless of the shape of the relationship of cAIx disagreement relative to average cAIx between different age decades, the variability of disagreement seems to be reduced as the individual data in a particular age cluster are closer to the corresponding regression line. This means that sensitivity of normative equations for detecting vascular phenotypes at risk could be improved if designed for narrower subsets of population in terms of the age, since the age is the most important determinant of the cAIx. Consequently, the comparability of such normative equations between different populations would also be facilitated and more reliable.

The proposed concept would be additionally strengthened if the number of participants in some strata was higher, which is an obvious weakness of our study. Nevertheless, the results were internally consistent as slopes of regression lines between age strata other than for men aged 30–39 and women aged 40–49 years were comparable, with a good-quality model fit (R^2^ from 0.44 to 0.70) and significant regression coefficients (P ≤ 0.001).

Possible differences in the sampling frame in terms of the age distribution between ours and the Danish low risk cohort, is another topic that needs to be addressed. As rightfully pointed out in the study of Chirinos *et al.*^14^ and in the associated commentary by Chowienczyk^6^, a different sampling frame might influence derived normative equations. In our previous work, we also recognized that homogeneity of the age of the study sample should be considered when interpreting results of studies investigating arterial stiffness indices^15^. Although no data on age distribution in the Danish low risk cohort are available, we cannot exclude sampling frame differences between the two cohorts. However, it appears that there was no relevant difference as regression coefficients of the original equations and the equations fitted onto Croatian cohort did not differ (Table 2).

In addition, the results of this study should be considered strictly to be device specific. Namely, it has been repeatedly shown that PWA data obtained with different devices employing different measuring principles cannot be interchangeably used^15^,^21^,^22^. Despite the available statistical tools and recommendations on the ways of comparing values from different devices, such practice may result in multiplication of errors and increased noise in the dataset^23^.

Taken together, it can be concluded that the impact of the differences between Nordic and Mediterranean environments on the vascular phenotypes in the low CVD risk populations appears not to be significant. The proposed normative equations, however, allow large inter-individual variability in cAIx values and are not equally applicable across age ranges. We propose that normative equations should be designed for narrower age groups and they should include additional determinant(s) of cAIx besides age, sex, height, and heart rate. That would increase the sensitivity of the equation in detecting vascular phenotypes at risk, facilitate the comparability between different populations, and practically remove the impact of difference in the sampling frame.

## Subjects and Methods

### Study design and sampling

This cross-sectional study is based on a general population sample of 1,008 adult volunteers (≥18 years of age) from the coastal city of Split (Croatia) and the surrounding area. This sample size was sufficient for a survey estimating the target population parameters with up to a 3.5% error, given the confidence level of 95% and the population size. The study was conducted from October 2008 to December 2009 at the University of Split School of Medicine as a part of the large population-based survey “10,001 Dalmatians”^24^. Subjects were invited to participate in the study according to the telephone directory. Those who expressed willingness to take part in the study were scheduled for a visit to the medical school, where research coordinators initially explained the aims and goals of the project, followed by the informed consent signing, and then measurements of various parameters. Each participant completed a detailed medical history and standardized questionnaires on their lifestyle, habits, exposure to health risks, and health attitudes. Participants were subjected to a thorough physical examination that included anthropometric measurements, assessments of the blood pressure and the pulse wave analysis (PWA). Fasting blood samples were taken for standard biochemical analysis including levels of glucose, triglycerides, LDL, HDL, and total cholesterol.

The study complies with the Declaration of Helsinki, was approved by the Ethics Committee of the University of Split School of Medicine, and all participants provided written informed consent.

### Selection of Croatian low risk cohort

Participants included in the reference data set were selected from the initial number of 1,008 participants with successfully recorded cAIx. After applying the identical set of eligibility criteria from the study by Janner *et al.* on the Danish general population-based sample^13^, the final Croatian reference set included 538 (53%) participants.

The selection steps were as follows: firstly, we excluded 89 participants with a history of diabetes or cardiovascular diseases (CVD). Afterwards, we estimated the risk of CVD for all remaining participants by applying the HeartScore — the electronic counterpart of the SCORE risk charts published by the European Guidelines on Cardiovascular Prevention^25^. This score system uses data on age, sex, total cholesterol, systolic blood pressure, and smoking habits to estimate the risk of CVD for the next 10 years. We could not assess the HeartScore for 15 subjects because of missing data so we excluded them from subsequent analysis.

The remaining 904 subjects were further divided by sex and stratified into age decades from 20 years to 80 years and older. In each age-group, the participants were additionally divided into tertiles of the HeartScore. Those belonging to the lowest risk tertiles comprised the final Croatian reference set.

**Figure 6:**
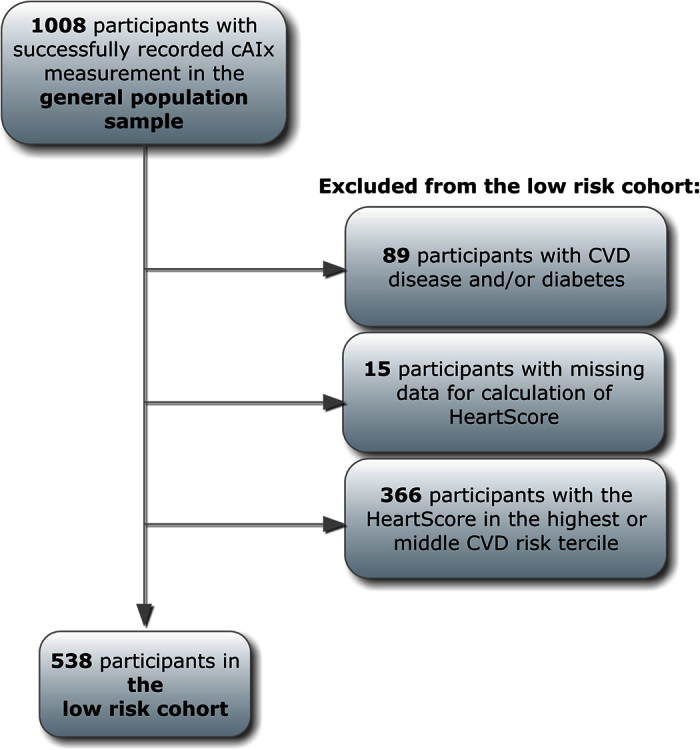
Study flow chart.

The described selection steps are shown in the study flow chart (Fig. 6).

### Hemodynamic measurements

The measurements were performed in accordance to the expert consensus document on arterial stiffness by the European Network for Non-Invasive Investigation of Large Arteries^3^. The participants were resting for 10 minutes in a supine position in a quiet and temperature-controlled laboratory (22 °C) before the right radial artery pulse waves were recorded non-invasively by applanation tonometry using the SphygmoCor apparatus (AtCor Medical, Inc., Sydney, Australia). Central hemodynamic indices including cAIx were derived using the Sphygmocor’s built-in generalized transfer function^26^. From each participant, two measurements that satisfied the device’s built-in quality control criteria were obtained. All measurements were performed by two well-trained operators.

Blood pressure was measured using an aneroid sphygmomanometer (Accoson model AC0332, Welwyn Garden City, UK). Two measurements of systolic and diastolic blood pressures were taken in each subject 5 minutes apart, and the mean value of the readings was used in the analysis.

### The Danish normative equations for cAIx applied on the Croatian low risk CVD cohort

Danish population-based normative equations developed by Janner *et al.*^13^ were used to calculate reference cAIx values in the selected Croatian low risk CVD cohort:


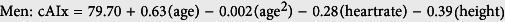



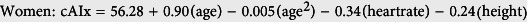


Normative equations allow a standardized comparison between individual cAIx measurements and the predicted, reference cAIx population mean adjusted for subject’s age, body height, and heart rate.

### Data analysis

In order to evaluate selection criteria and comparability between the Danish and Croatian cohorts, the actually measured individual cAIx values from the Croatian low risk cohort were portrayed on *cAIx-age* scatter chart and plotted against the 95% prediction limits curves taken from the corresponding chart on the Danish low risk cohort. The curves were extracted from the original publication with the Danish cohort^13^ by the automatic curve extraction algorithm WebPlotDigitizer which excerpt a large number of data points from images (URL: http://arohatgi.info/WebPlotDigitizer/app/).

BA plots, for both men and women, visualized agreement between the cAIx values predicted by the Danish normative equation and those actually measured in the Croatian low risk cohort. The best fitted BA curves were estimated as follows: a) a regression of the difference between observed and predicted cAIx values against their averages was assessed with both linear and nonlinear regression models and b) based on the curve estimation regression statistics, the best fit BA model was identified, and 95% limits-of-agreement curves around the fitted line were determined. We employed an analysis of residuals to estimate homoscedasticity, assessing the significance of the relationship between the standard deviation of the differences and the magnitude of the average cAIx with the F-test. In each sex, BA analysis was performed on the overall sample and on age subgroups: 20–29 (53 women and 33 men), 30–39 (59 women and 30 men), 40–49 (111 women and 24 men), 50–59 (122 women and 30 men), and 60–69 (33 women) years. The best fit BA models for the men 60–69 years of age (n = 18) as well as the 18–19 (3 women and 1 man) years and 70 years and older (9 women and 12 men) age subgroups were not built due to a smaller sample size.

In addition to the BA analysis, we performed the Pearson’s correlation analysis to examine the relationship between cAIx values predicted by the Danish normative equation and those observed in the Croatian low risk cohort.

To estimate the contribution of each cAIx determinant from the equations (age^2^, age, HR, and height) to the cAIx values observed in the low risk Croatian participants, we replicated the multiple regression model that was used by Janner *et al.* and fitted it onto data from the Croatian cohort. Differences in the contribution of the particular cAIx determinant between the two population samples were inferred from the 95% CI for unstandardized coefficient B.

All regression models presented in our study met the sample size recommendation for adequate regression modelling for both the whole low risk cohort and the age groups. Specifically, all the models had from 24 to 130 subjects per independent variable which satisfied commonly accepted rules-of-thumb recommendations^27^,^28^,^29^.

Differences in means of quantitative variables between the Croatian and Danish population samples were tested by the unpaired two-sample t-test, whereas the chi-square test was used to test differences in the frequency distribution of categorical variables.

As the proportion of missing data in this study was low (n = 15, 1.5%), a list wise deletion strategy based on the Heartscore availability was applied. All tests were two sided; the level of statistical significance was set at 5%, and all statistical calculations were done with IBM SPSS Statistics 19.0 software (SPSS Inc., Chicago, USA).

## Additional Information

**How to cite this article**: Jeroncic, A. *et al.* Normative equations for central augmentation index: assessment of inter-population applicability and how it could be improved. *Sci. Rep.* **6**, 27016; doi: 10.1038/srep27016 (2016).

## Figures and Tables

**Figure 1:**
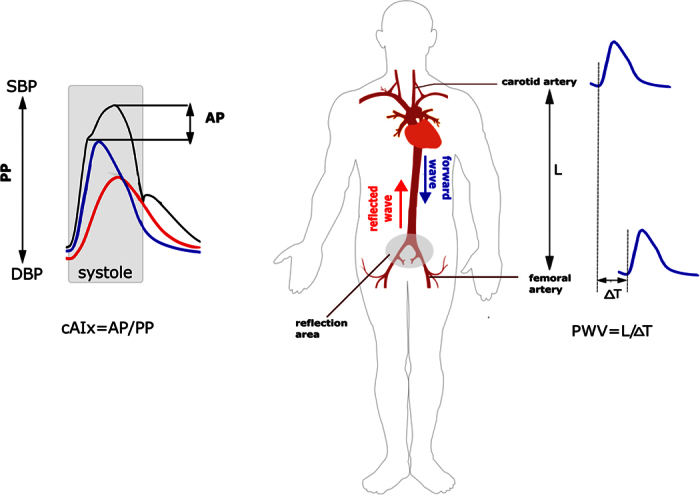
Principles of assessment of pulse wave velocity (PWV) and central augmentation index (cAIx) as indices of arterial stiffness. Right panel - The pulse wave generated by contraction of the left ventricle of the heart travels down the arterial tree. The PWV is directly related to the arterial stiffness and it can be assessed by measuring its travelling time between two recording sites along the analyzed arterial segment. Carotid-femoral PWV measurement is considered as a “gold standard” method; Left panel - The forward travelling pulse wave is reflected in the area of impedance mismatch where the elastic aorta branches into narrower muscular conduit arteries. The shape of the pressure waveform in the central aorta (black curve) is influenced by the overlap between forward (blue curve) and reflected pulse waves (red curve). With aortic stiffening PWV increases and the reflected wave returns earlier in systole (shaded) resulting in augmentation of the aortic systolic and pulse pressure. PWV - pulse wave velocity; AP - augmentation pressure; PP - pulse pressure; cAIx – central augmentation index; SBP - systolic blood pressure; DBP – diastolic blood pressure.

**Table 1:** Baseline characteristics of the total Croatian (n = 1008) and Danish (n = 3432) general population-based samples with successfully recorded cAIx measurements.

	Men, mean ± SD or n(%)			Women, mean ± SD or n(%)		
	Croatian, n = 394	Danish, n = 1490	Mean or Percentage Difference (95% CI)	Croatian, n = 614	Danish, n = 1942	Mean or Percentage
Age (years)	49.3 ± 15.4	57.7 ± 15.8	−8.4 (−10.1, −6.7)	51.0 ± 13.7	59.7 ± 16.4	−8.7 (−10.1, −7.3)
Height (cm)	180.8 ± 6.4	176.9 ± 7.4	3.9 (3.1, 4.7)	166.7 ± 6.3	163.8 ± 7.0	2.9 (2.3, 3.5)
Weight (kg)	92.1 ± 14.2	82.7 ± 12.6	9.4 (8.0, 10.8)	73.0 ± 13.2	68.5 ± 12.7	4.5 (3.3, 5.7)
SBP (mmHg)	130.3 ± 15.6	139.1 ± 20.5	−8.8 (−11.0, −6.6)	122.8 ± 17.6	138.8 ± 24.4	−16.0 (−18.1, −13.9)
DBP (mmHg)	78.2 ± 12.3	80.6 ± 11.7	−2.4 (−3.7, −1.1)	73.2 ± 12.1	77.9 ± 11.7	−4.7 (−5.8, −3.6)
Total cholesterol (mmol/L)	5.8 ± 1.3	5.4 ± 1.2	0.4 (0.2, 0.5)	5.9 ± 1.2	5.6 ± 1.2	0.3 (0.2, 0.4)
LDL (mmol/L)	3.8 ± 1.1	3.5 ± 1.0	0.3 (0.2, 0.4)	3.9 ± 1.1	3.5 ± 1.1	0.4 (0.3, 0.5)
HDL (mmol/L)	1.2 ± 0.3	1.3 ± 0.4	−0.1 (−0.14, −0.06)	1.5 ± 0.3	1.6 ± 0.5	−0.1 (−0.14, −0.06)
Triglycerides (mmol/L)	1.8 ± 1.3	1.7 ± 1.8	0.1 (−0.01, 0.3)	1.3 ± 0.67	1.4 ± 0.8	−0.1 (−0.17, −0.03)
Smoking						
Smokers	95 (24.1)	514 (34.5)	−10.4% (−15.0, −0.1)	170 (27.7)	727 (37.0)	−9.7% (−13.8, −5.5)
Never-smokers	158 (40.1)	389 (26.1)	13.9% (8.8, 19.3)	306 (49.8)	609 (31.0)	18.5% (14.0, 23.0)
Ex-smokers	141 (35.8)	558 (37.5)	−1.6% (−2.6, 1.1)	138 (22.5)	585 (30.1)	−7.6% (−11.4, −3.6)
Stroke	9 (2.3)	50 (3.4)	−1.1% (−1.5, 2.5)	7 (1.1)	37 (1.9)	−0.8% (−1.7, 0.5)
Diabetes	22 (5.6)	94 (6.3)	−0.7% (−3.0, 2.2)	23 (3.7)	71 (3.7)	<0.1% (−1.5, 2.1)
IHD	19 (4.8)	124 (8.3)	−3.5% (−5.8, −0.6)	21 (3.4)	98 (5.1)	−1.6% (−3.2, 0.3)
cAIx (%)	16.82 ± 14.25	21.80 ± 12.50	−5.00 (−6.4, −3.6)	25.01 ± 13.48	30.00 ± 11.80	−5.0 (−6.1, −3.9)

Abbreviations: SBP- systolic blood pressure; DBP - diastolic blood pressure; IHD - ischemic heart disease; cAIx – central augmentation index.

**Table 2:** The Danish normative equations for cAIx applied on the low risk Croatian cohort.

Sex	cAIx determinant	The Danish normative equations	The Danish normative equation applied on the Croatian cohort	
		B coefficient (95% CI)	B coefficient (95% CI)	R^2^
Men	Age [years]	0.63 (0.34 to 0.92)	0.64 (0.53 to 0.75)	0.56
	Age^2^ [years]	−0.002 (−0.005 to −0.0004)	−0.008 (−0.014 to −0.001)	
	HR [beats/min]	−0.28 (−0.36 to −0.19)	−0.38 (−0.55 to −0.20)	
	Height [cm]	−0.39 (−0.52 to −0.26)	−0.23 (−0.48 to 0.03)	
Women	Age [years]	0.90 (0.64 to 1.16)	0.63 (0.56 to 0.70)	0.58
	Age^2^ [years]	−0.005 (−0.017 to −0.003)	−0.012 (−0.017 to −0.008)	
	HR [beats/min]	−0.34 (−0.42 to −0.26)	−0.40 (−0.50 to −0.30)	
	Height [cm]	−0.24 (−0.36 to −0.12)	−0.37 (−0.50 to −0.23)	

The regression coefficients from the original and the fitted equation are compared. Differences in contribution of the particular cAIx determinant were estimated from 95% CI for unstandardized coefficient B.
